# Supplementary material for: Contribution of climate change to the spatial expansion of West Nile virus in Europe
Source: Nat Commun. 2024 Feb 8;15:1196. doi: 10.1038/s41467-024-45290-3 (PMC10853512; doi:10.1038/s41467-024-45290-3)
Supplement: Supplementary file 1 — Supplementary Information [file 41467_2024_45290_MOESM1_ESM.pdf]

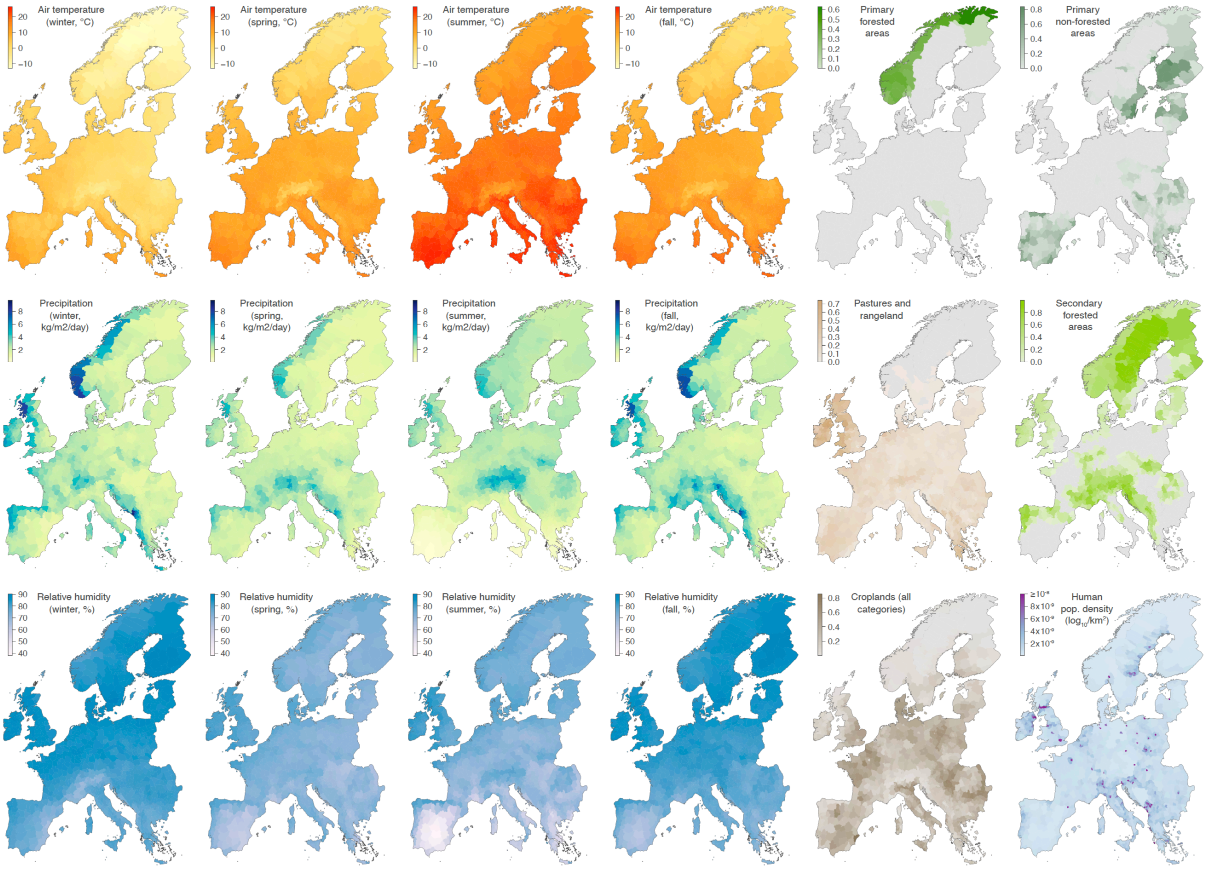

Figure S1. Environmental factors included in the ecological niche modelling analyses of WNV in Europe (2000-2019). We here display, for each optimised NUTS3 administrative area, the environmental values extracted from the ISIMIP3a reanalysis dataset GSWP3-W5E5.

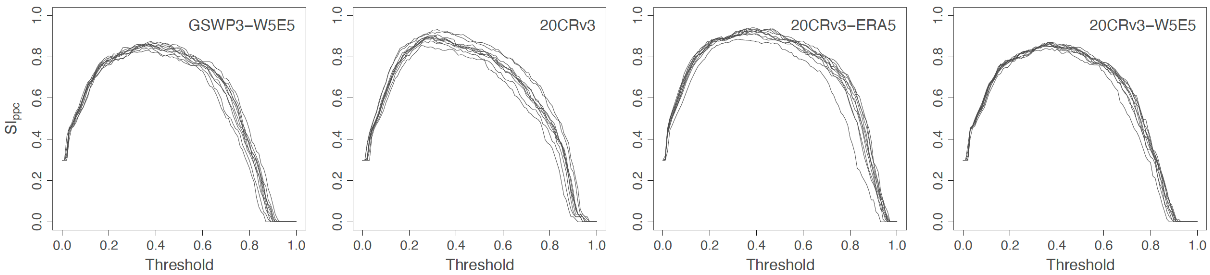

Figure S2. Assessment of the predictive performance of ecological niche models based on the computation of the prevalence-pseudoabsence-calibrated Sørensen index (SI_{ppc}). Specifically, we computed the SI_{ppc} while performing an optimisation of the ecological suitability threshold in the range [0, 1] with a 0.01 step increment. This threshold value was used to generate binary versions of the ecological suitability maps necessary for the computation of this index, and we eventually selected the threshold value maximising the SI_{ppc} (see Table S1 for the optimised SI_{ppc} values and associated threshold).

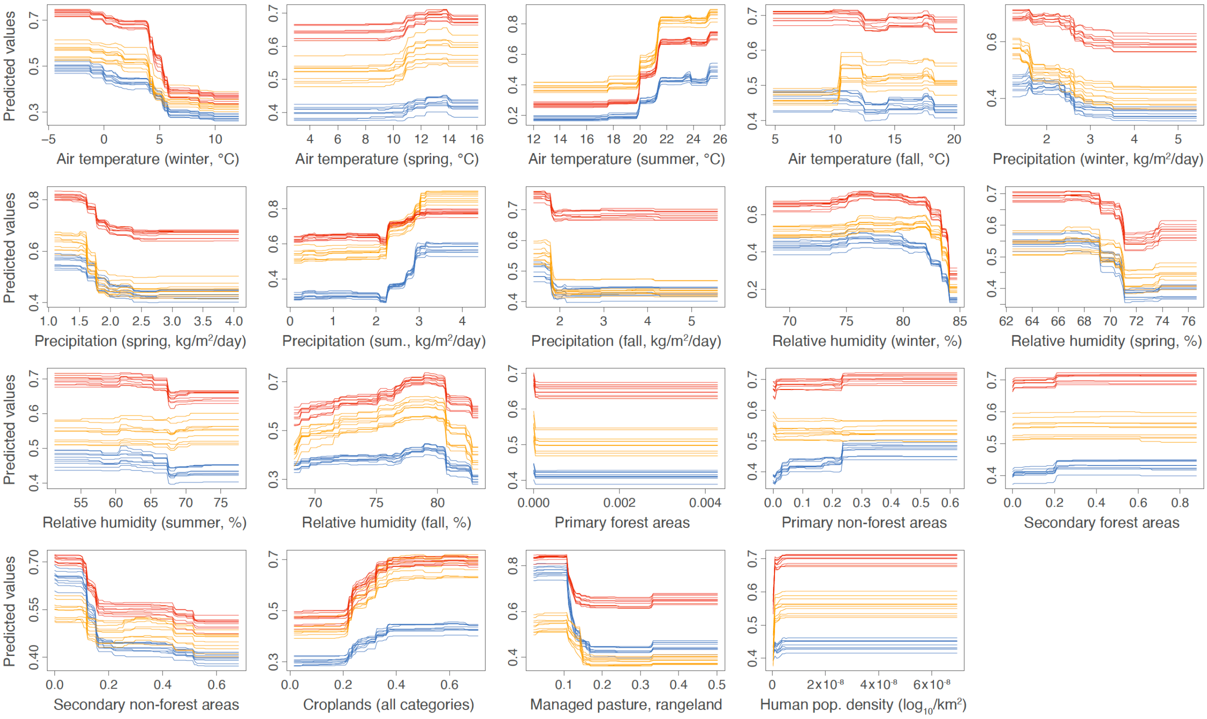

Figure S3. Responses curves of the ecological niche modelling. For each environmental factor, we report the response curve of each of the ten replicate boosted regression tree (BRT) models trained on present-day data retrieved from the ISIMIP3a reanalysis dataset GSWP3-W5E5. These response curves indicate the relationship between the environmental values and the response, i.e. the ecological suitability of WNV, and were obtained by computing the ecological suitability variation associated with one specific variable, while all others were kept constant at their median (red curves), first quartile (orange curves) or third quartile (blue curves) value.

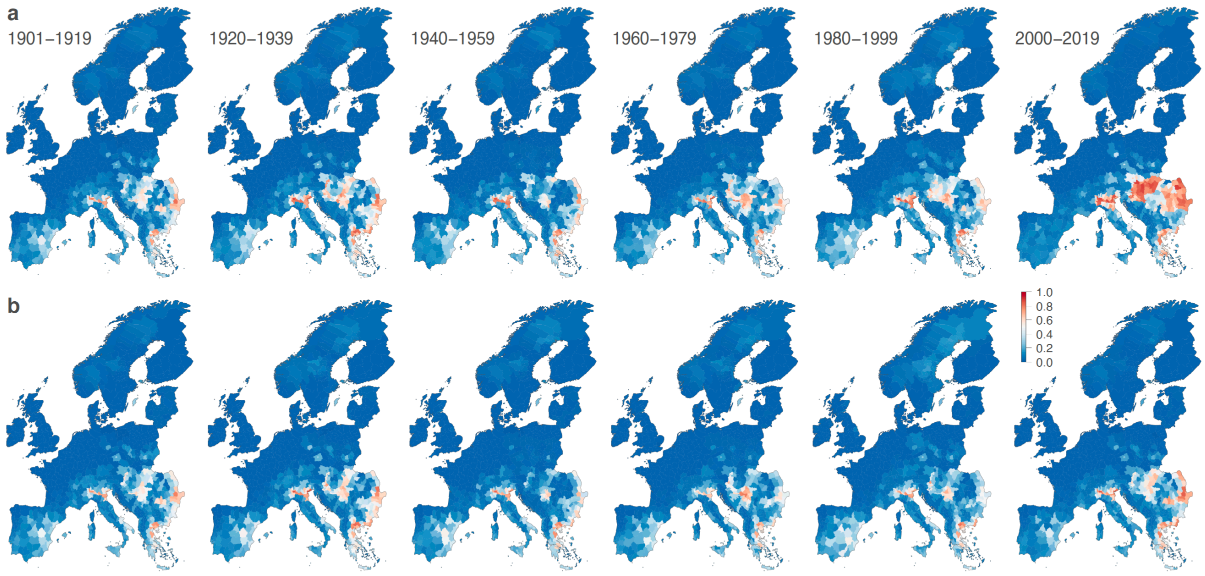

Figure S4. Changes in the ecological suitability of WNV in Europe based on data retrieved from the 20CRv3-W5E5 reanalysis dataset. Past and present ecological suitability estimated for each administrative unit are based on both the reconstructions of the historical climate (**a**) and a counterfactual baseline (**b**). Ecological suitability values are averaged over the estimates of ten independent BRT models trained on present-day data retrieved from the ISIMIP3a reanalysis dataset 20CRv3-W5E5.

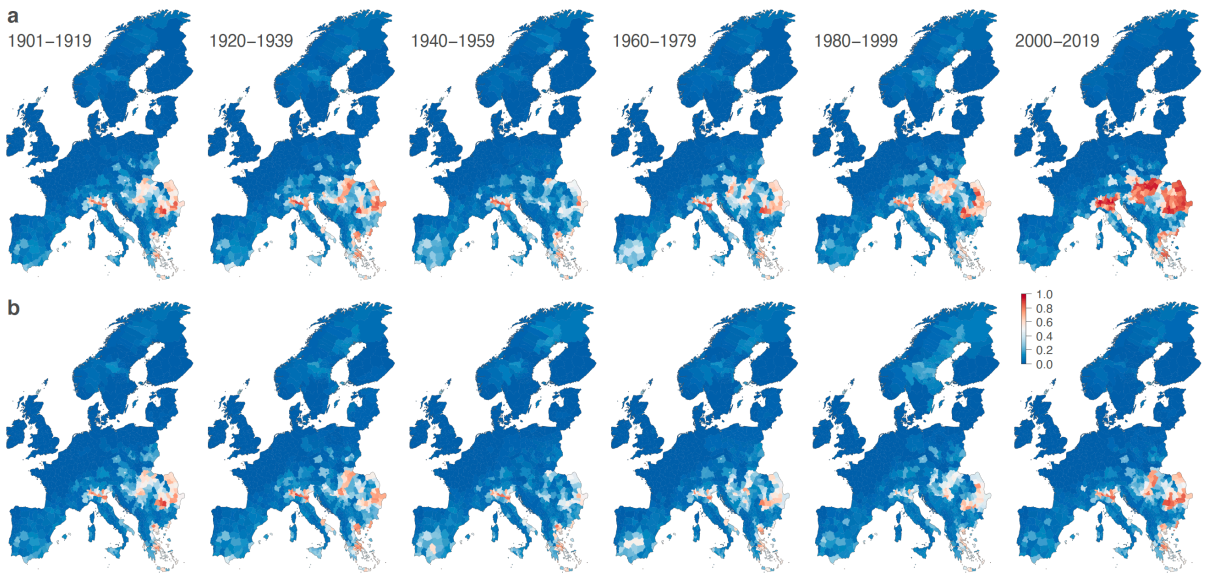

Figure S5. Changes in the ecological suitability of WNV in Europe based on data retrieved from the 20CRv3-ERA5 reanalysis dataset. Past and present ecological suitability estimated for each administrative unit are based on both the reconstructions of the historical climate (**a**) and a counterfactual baseline (**b**). Ecological suitability values are averaged over the estimates of ten independent BRT models trained on present-day data retrieved from the ISIMIP3a reanalysis dataset 20CRv3-ERA5.

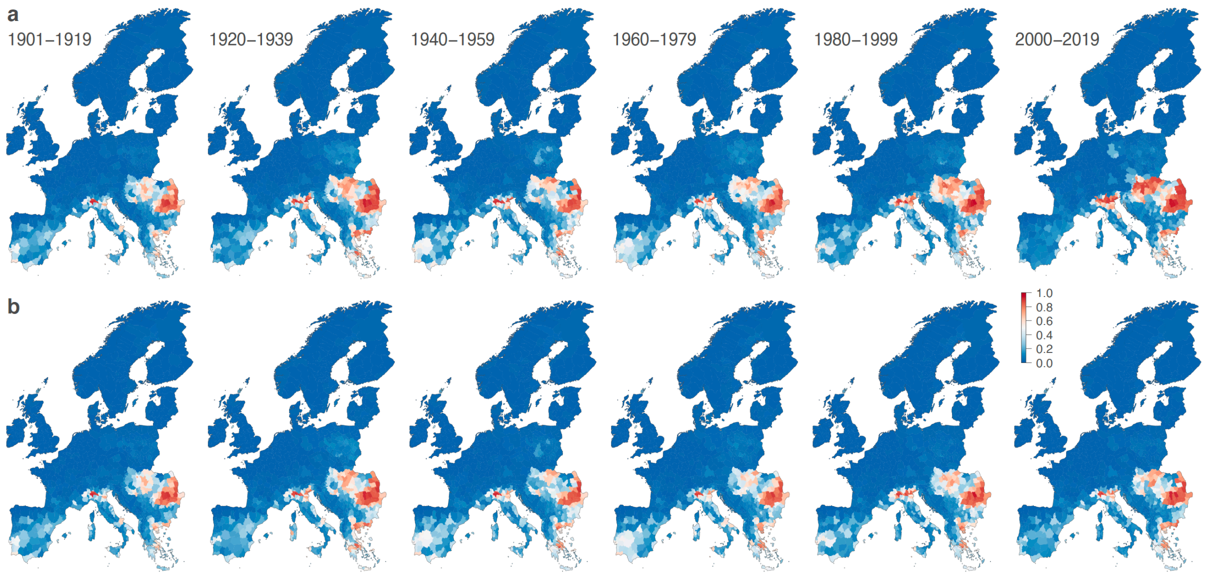

Figure S6. Changes in the ecological suitability of WNV in Europe based on data retrieved from the 20CRv3 reanalysis dataset. Past and present ecological suitability estimated for each administrative unit are based on both the reconstructions of the historical climate (**a**) and a counterfactual baseline (**b**). Ecological suitability values are averaged over the estimates of ten independent BRT models trained on present-day data retrieved from the ISIMIP3a reanalysis dataset 20CRv3.

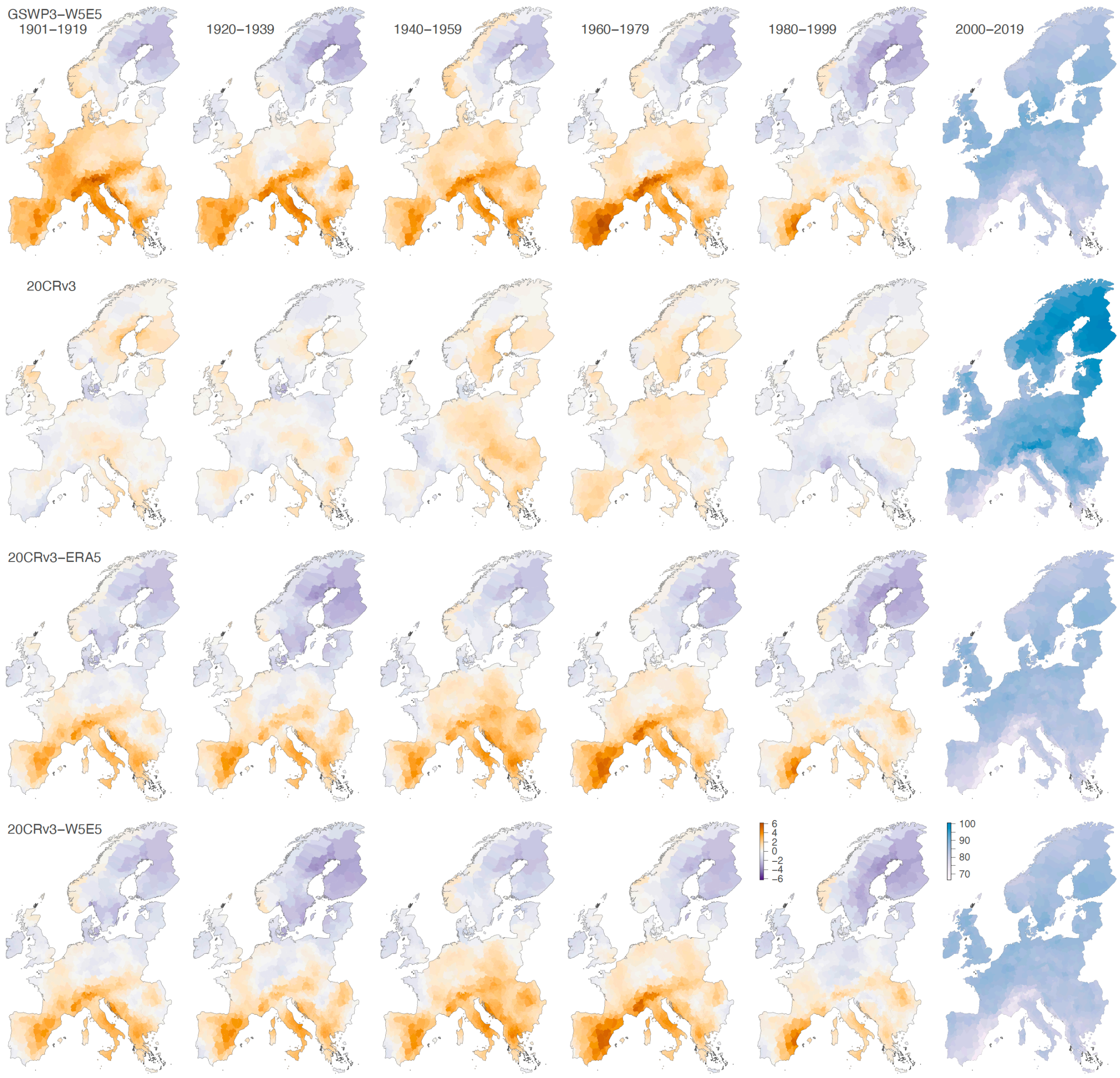

Figure S7. Past changes in relative humidity in winter according to the four ISIMIP3a reanalysis datasets. Each column of maps corresponds to a specific period, and each row of maps corresponds to one of the four considered ISIMIP3a reanalysis datasets. For the period 2000-2019, we report the absolute values (% of relative humidity in winter), and for all the previous periods, we report the difference with the period 2000-2019.

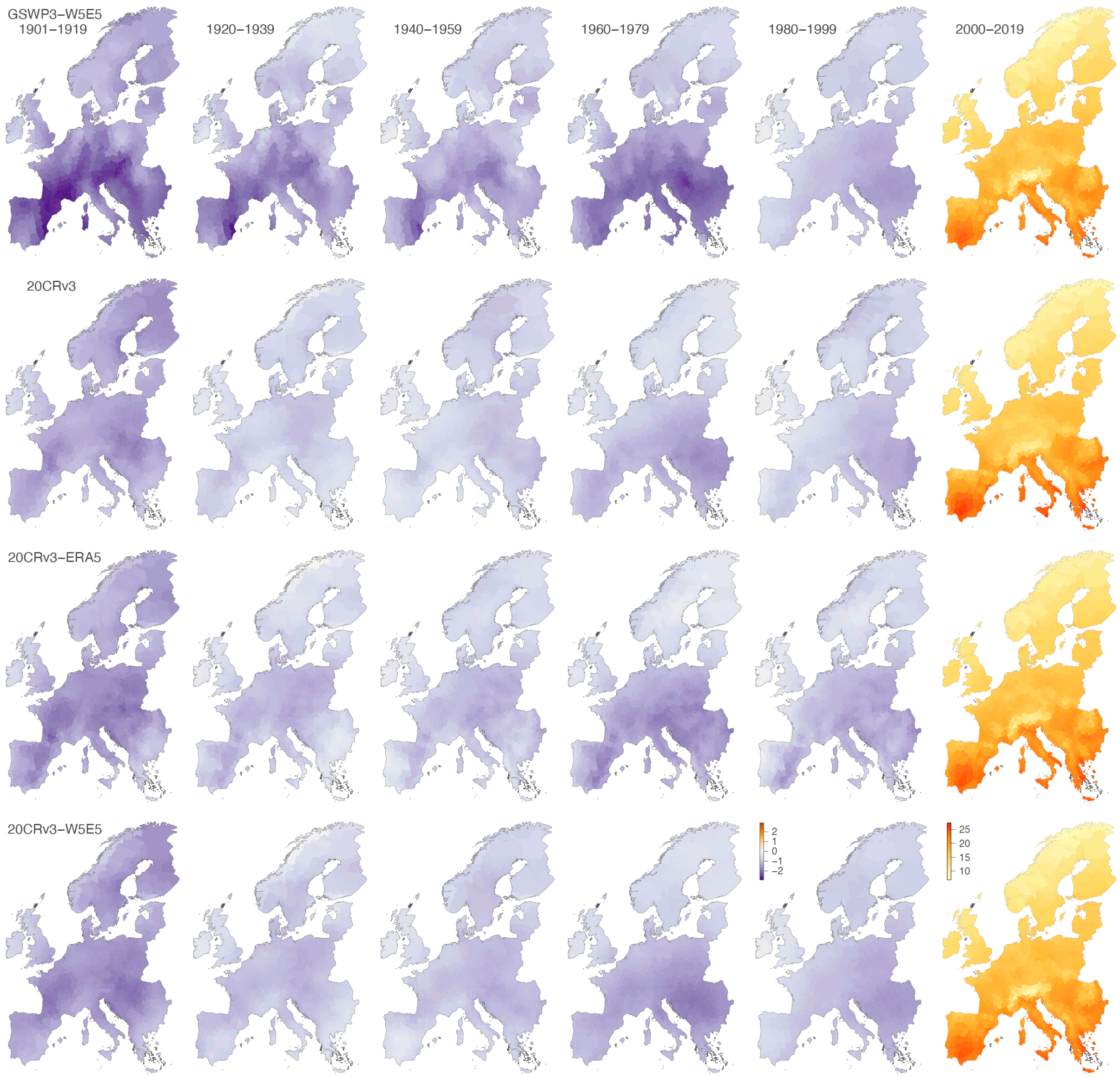

Figure S8. Past changes in air temperature in summer according to the four ISIMIP3a reanalysis datasets. Each column of maps corresponds to a specific period, and each row of maps corresponds to one of the four considered ISIMIP3a reanalysis datasets. For the period 2000-2019, we report the absolute values (°C in summer), and for all the previous periods, we report the difference with the period 2000-2019.

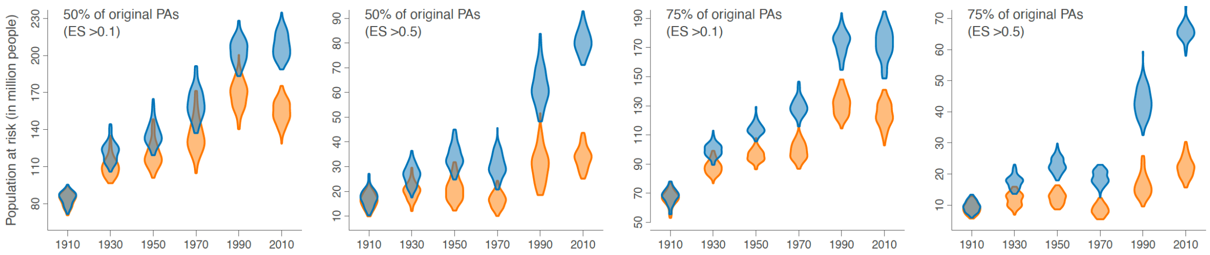

Figure S9. Investigations of the robustness and sensitivity of the estimates of human population at risk of exposure to the sampling intensity of pseudo-absences across the study area. Similar to Figure 3, we here report past changes in the population at risk of exposure to WNV estimated for both a counterfactual baseline and the observed historical climate data retrieved from the GSWP3-W5E5 reanalysis dataset and while considering two different thresholds of ecological suitability (ES) above which an area was considered at risk (0.1 and 0.5). Contrary to Figure 3, the estimates reported here are however based on ecological niche models trained on random subsets of all available pseudo-absences (PAs) corresponding to all optimised NUTS3 administrative areas with zero confirmed non-imported human cases of WNV infection. Specifically, we considered both a subsampling of 50 and 75% of all pseudo-absence data and, for each percentage, re-trained 100 ecological ecological niche models each time based on a random subset of pseudo-absence data.

Table S1. Predictive performance of ecological niche models. The table reports area under the curve (AUC) and prevalence-pseudoabsence-calibrated Sørensen index (SI_{ppc}) values computed for each ecological niche model trained in the present study with a spatial cross-validation approach. Specifically, we trained ten independent replicate boosted regression tree (BRT) models on present-day data retrieved from each ISIMIP3a reanalysis datasets considered in our study (GSWP3-W5E5, 20CRv3, 20CRv3-ERA5, and 20CRv3-W5E5). The SI_{ppc} were computed while performing an optimisation of the ecological suitability threshold in the range [0, 1] with a 0.01 step increment. This threshold value was used to generate binary versions of the ecological suitability maps necessary for the computation of this index, and we eventually selected the threshold value maximising the SI_{ppc}, which is here reported under parentheses.

	**Area under the curve (AUC)**				**SI_{ppc} (and threshold value maximising SI_{ppc})**			
	**GSWP3-W5E5**	**20CRv3**	**20CRv3-ERA5**	**20CRv3-W5E5**	**GSWP3-W5E5**	**20CRv3**	**20CRv3-ERA5**	**20CRv3-W5E5**
Replicate 1	0.87	0.86	0.86	0.85	0.86 (0.37)	0.90 (0.29)	0.91 (0.34)	0.87 (0.36)
Replicate 2	0.87	0.86	0.89	0.84	0.86 (0.37)	0.90 (0.31)	0.91 (0.37)	0.85 (0.35)
Replicate 3	0.85	0.80	0.83	0.85	0.85 (0.39)	0.85 (0.25)	0.88 (0.32)	0.86 (0.37)
Replicate 4	0.87	0.84	0.89	0.86	0.86 (0.37)	0.93 (0.32)	0.94 (0.38)	0.86 (0.34)
Replicate 5	0.84	0.82	0.87	0.84	0.86 (0.38)	0.89 (0.27)	0.93 (0.35)	0.87 (0.38)
Replicate 6	0.85	0.81	0.87	0.84	0.87 (0.38)	0.88 (0.29)	0.93 (0.39)	0.87 (0.37)
Replicate 7	0.85	0.81	0.87	0.85	0.83 (0.37)	0.92 (0.34)	0.93 (0.38)	0.84 (0.38)
Replicate 8	0.86	0.81	0.89	0.83	0.87 (0.38)	0.91 (0.32)	0.94 (0.46)	0.85 (0.39)
Replicate 9	0.83	0.82	0.88	0.86	0.84 (0.38)	0.89 (0.30)	0.92 (0.42)	0.86 (0.34)
Replicate 10	0.86	0.80	0.88	0.84	0.86 (0.35)	0.90 (0.31)	0.93 (0.41)	0.86 (0.37)

Table S2. Relative influence of environmental factors in the ecological niche models. The table reports the relative influence (RI, in %) of environmental variables averaged over the ten independent replicate boosted regression tree (BRT) models trained on present-day data retrieved from each ISIMIP3a reanalysis dataset considered in our study (GSWP3-W5E5, 20CRv3, 20CRv3-ERA5, and 20CRv3-W5E5). Under parentheses, we also report the minimum and maximum values associated with one replicate BRT model. The results obtained for environmental factors associated with an averaged RI value >10% are highlighted in bold.

	**GSWP3-W5E5**	**20CRv3**	**20CRv3-ERA5**	**20CRv3-W5E5**
Air temperature (winter)	**10.5 [10.1-11.1]**	8.0 [7.5-8.6]	5.0 [4.4-5.5]	**10.4 [9.9-11.1]**
Air temperature (spring)	1.4 [1.1-1.8]	2.5 [1.8-3.1]	1.2 [0.9-1.4]	1.5 [1.3-2.1]
Air temperature (summer)	**16.3 [15.8-16.7]**	**17.2 [16.3-17.8]**	**20.1 [19.6-20.6]**	**16.2 [15.6-16.7]**
Air temperature (fall)	1.8 [1.0-2.4]	3.6 [3.3-3.9]	2.1 [1.7-2.6]	1.8 [1.4-2.1]
Precipitation (winter)	5.8 [5.5-6.0]	**11.7 [11.4-11.8]**	**10.3 [10.0-10.8]**	5.9 [5.6-6.2]
Precipitation (spring)	6.2 [5.7-6.6]	3.9 [3.3-4.4]	2.5 [2.3-2.8]	6.2 [5.9-6.4]
Precipitation (summer)	8.5 [8.1-8.7]	8.3 [7.7-8.9]	**12.7 [12.4-13.1]**	8.3 [7.8-8.6]
Precipitation (fall)	1.9 [1.7-2.3]	4.8 [4.0.4-5]	3.5 [3.2-3.8]	1.9 [1.4-2.3]
Relative humidity (winter)	**10.1 [9.6-10.7]**	0.9 [0.7-1.3]	9.8 [9.3-10.2]	**10.3 [10.1-10.7]**
Relative humidity (spring)	6.3 [6.0-7.0]	3.1 [2.8-3.5]	8.1 [7.5-8.8]	6.3 [5.8-7.0]
Relative humidity (summer)	1.0 [0.6-1.3]	4.6 [4.2-4.9]	0.9 [0.6-1.1]	1.0 [0.9-1.2]
Relative humidity (fall)	7.5 [6.7-8.0]	7.8 [7.4-8.6]	6.7 [6.1-7.1]	7.4 [6.4-8.3]
Primary forest areas	0.5 [0.3-0.6]	0.7 [0.7-0.8]	0.1 [0.1-0.2]	0.5 [0.4-0.6]
Primary non-forest areas	2.3 [1.9-2.6]	2.8 [2.6-3.1]	2.3 [2.0-2.5]	2.3 [1.8-2.8]
Secondary forest areas	0.4 [0.3-0.5]	0.2 [0.1-0.3]	0.1 [0.1-0.2]	0.4 [0.3-0.5]
Secondary non-forest areas	4.8 [4.5-5.3]	3.5 [3.2-3.7]	1.9 [1.6-2.1]	4.8 [4.4-5.3]
Croplands (all categories)	4.7 [4.5-4.9]	5.8 [5.0.6-6]	4.2 [3.9-4.8]	4.8 [4.5-5.1]
Managed pastures, rangeland	7.1 [6.7-7.3]	7.9 [7.5-8.2]	5.5 [5.1-5.8]	7.1 [6.7-7.5]
Human pop. density (log_{10}/km^2)	2.7 [2.4-3.1]	2.9 [2.5-3.2]	2.8 [2.0.4-3]	2.8 [2.6-3.2]

Table S3. Sensitivity of the area under the receiving operator curve (AUC) support to the specification of the tree complexity and learning rate BRT parameters. For each combination of tested tree complexity and learning rate parameter values, we report the median AUC support [as well as the first and third quartiles] obtained across ten replicates based on the GSWP3-W5E5 reanalysis dataset.

**Tree complexity**	**Learning rate**				
	**0.0005**	**0.001**	**0.005**	**0.01**	**0.05**
**1**	0.84 [0.84-0.85]	0.86 [0.86-0.87]	0.88 [0.87-0.88]	0.88 [0.88-0.89]	0.88 [0.88-0.89]
**5**	0.89 [0.89-0.90]	0.90 [0.89-0.91]	0.90 [0.90-0.91]	0.90 [0.90-0.91]	-
**10**	0.91 [0.90-0.91]	0.91 [0.91-0.92]	0.91 [0.91-0.92]	0.91 [0.90-0.92]	-
