# Supplementary material for: Contribution of climate change to the spatial expansion of West Nile virus in Europe
Source: Nat Commun. 2024 Feb 8;15:1196. doi: 10.1038/s41467-024-45290-3 (PMC10853512; doi:10.1038/s41467-024-45290-3)
Supplement: Supplementary file 3 — Reporting Summary [file 41467_2024_45290_MOESM3_ESM.pdf]

nature portfolio

Corresponding author(s): Diana Erazo, Simon Dellicour

Last updated by author(s): 03/01/2024

Reporting Summary

Nature Portfolio wishes to improve the reproducibility of the work that we publish. This form provides structure for consistency and transparency in reporting. For further information on Nature Portfolio policies, see our Editorial Policies and the Editorial Policy Checklist.

Statistics

For all statistical analyses, confirm that the following items are present in the figure legend, table legend, main text, or Methods section.

n/a	Confirmed	
☐	☒	The exact sample size (n) for each experimental group/condition, given as a discrete number and unit of measurement
☒	☐	A statement on whether measurements were taken from distinct samples or whether the same sample was measured repeatedly
☒	☐	The statistical test(s) used AND whether they are one- or two-sided<br>*Only common tests should be described solely by name; describe more complex techniques in the Methods section.*
☐	☒	A description of all covariates tested
☐	☒	A description of any assumptions or corrections, such as tests of normality and adjustment for multiple comparisons
☐	☒	A full description of the statistical parameters including central tendency (e.g. means) or other basic estimates (e.g. regression coefficient) AND variation (e.g. standard deviation) or associated estimates of uncertainty (e.g. confidence intervals)
☒	☐	For null hypothesis testing, the test statistic (e.g. F, t, r) with confidence intervals, effect sizes, degrees of freedom and P value noted<br>*Give P values as exact values whenever suitable.*
☒	☐	For Bayesian analysis, information on the choice of priors and Markov chain Monte Carlo settings
☒	☐	For hierarchical and complex designs, identification of the appropriate level for tests and full reporting of outcomes
☒	☐	Estimates of effect sizes (e.g. Cohen's d, Pearson's r), indicating how they were calculated

Our web collection on statistics for biologists contains articles on many of the points above.

Software and code

Policy information about availability of computer code

Data collection	No software was used for data collection.
Data analysis	For the ecological niche modelling we used the boosted regression trees (BRT) algorithm implemented in the R package “dismo” (version 1.3-9). To account for spatial autocorrelation, we implemented a spatial cross-validation procedure based on the blocks generation described by Valavi and colleagues and implemented in the R package “blockCV” (version 3.1-1; Valavi et al. 2019). All analyses were performed using R (R Statistical Software version 4.02, R Foundation for Statistical Computing, https://www.r-project.org/). R scripts related to the ecological niche modelling are all available at https://github.com/sdellicour wnv_enm_europe.

For manuscripts utilizing custom algorithms or software that are central to the research but not yet described in published literature, software must be made available to editors and reviewers. We strongly encourage code deposition in a community repository (e.g. GitHub). See the Nature Portfolio guidelines for submitting code & software for further information.

Data

Policy information about availability of data

All manuscripts must include a data availability statement. This statement should provide the following information, where applicable:

- Accession codes, unique identifiers, or web links for publicly available datasets
- A description of any restrictions on data availability
- For clinical datasets or third party data, please ensure that the statement adheres to our policy

R scripts related to the ecological niche modelling are all available at https://github.com/sdellicour/wnv_enm_europe. All ISIMIP3a data used are publicly available on https://data.isimip.org/. Data on WNV human infections can be obtained on request from The European Surveillance System (TESSy) at the European Center for Disease Prevention and Control (ECDC) on https://www.ecdc.europa.eu/en/publications-data/european-surveillance-system-tessy.

Research involving human participants, their data, or biological material

Policy information about studies with human participants or human data. See also policy information about sex, gender (identity/presentation), and sexual orientation and race, ethnicity and racism.

Reporting on sex and gender	N/A
Reporting on race, ethnicity, or other socially relevant groupings	N/A
Population characteristics	N/A
Recruitment	N/A
Ethics oversight	N/A

Note that full information on the approval of the study protocol must also be provided in the manuscript.

Field-specific reporting

Please select the one below that is the best fit for your research. If you are not sure, read the appropriate sections before making your selection.

☐ Life sciences ☐ Behavioural & social sciences ☒ Ecological, evolutionary & environmental sciences

For a reference copy of the document with all sections, see nature.com/documents/nr-reporting-summary-flat.pdf

Ecological, evolutionary & environmental sciences study design

All studies must disclose on these points even when the disclosure is negative.

Study description	We performed ecological niche modeling to investigate the contribution of climate change to West Nile virus (WNV) spatial expansion in Europe. Specifically, we used a boosted regression tree (BRT) approach to estimate the WNV ecological suitability as an impact indicator of WNV infection risk, and assessed its performance for the historical period and compare it to a counterfactual impact baseline. We used four observational climate datasets and their counterfactuals that were recently made available through the Inter-Sectoral Impact Model Intercomparison Project (ISIMIP). The input data were twofold: 13 years of confirmed WNV human infections across the European continent for the period (2007-2019), as well as climatic, land use, and human population data for a corresponding period (2000-2019). We trained ten replicate models on such present-day data retrieved from four ISIMIP3a reanalysis datasets of historical climate change (GSWP3-W5E5, 20CRv3, 20CRv3-ERA5, and 20CRv3-W5E5). We subsequently estimated the past dynamics of the WNV ecological niche since the beginning of the 20th century considering either the historical climate or its respective counterfactual. Since the simulated historical WNV ecological suitability notably differs among the four ISIMIP3a reanalysis datasets used to train the models, we did not average their outcome and independently reported the simulations based on each dataset considered, which allowed pointing and discussing their differences.
Research sample	WNV human infection records in Europe aggregated at the NUTS level 3 (NUTS3) from 2007 to 2019 were retrieved and curated from The European Surveillance System (TESSy) database of the European Center for Disease prevention and control (ECDC). This dataset therefore represented the population infected with WNV in Europe. Climate, land use, and population data were retrieved from the Inter- Sectoral Impact Model Intercomparison Project phase 3 (ISIMIP3, https://www.isimip.org/protocol/3/). ISIMIP prescribes protocols and background datasets for modelling the impacts of climate change in various systems sensitive to climate and human management. In ISIMIP3a, modellers run historical impact simulations with reanalysis datasets, which are global reconstructions of the historical climate. Importantly, these datasets are provided additionally in a counterfactual version without the climate change signal to enable model evaluation and attribution of the impacts of climate change. Land use data were retrieved from the Land Use Harmonisation project (version 2; LUH2) providing historical and projected land use states. Land use data included six land cover categories: primary forest areas, primary non-forest areas, secondary forest areas, secondary non-forest areas, croplands, and rangelands/pastures.

Sampling strategy	NUTS3 is the best spatial resolution provided by the European Center for Disease prevention and control (ECDC) and was considered the spatial unit of the study. However, the sizes of NUTS3 polygons vary considerably among European countries. For example, NUTS3 level polygons for Germany are relatively small and similar in size to NUTS4 of other countries. We therefore used an optimised NUTS map in which levels were chosen to homogenise as much as possible the polygon unit size and defined the study sample size. This standard shapefile was developed for the European network for medical and veterinary entomology (VectorNet), a project led by the ECDC and the European Food Safety Authority (EFSA) that aims to contribute to improving preparedness and response for vector-borne pathogens following a one health approach. Therefore, WNV human infection records were aggregated following the optimised NUTS map polygons. Climate information used in this study from four ISIMIP3a reanalysis datasets of historical climate change (GSWP3-W5E5, 20CRv3, 20CRv3-ERA5, and 20CRv3-W5E5), consists of daily gridded near-surface air temperature, surface precipitation and relative humidity. Climate data, i.e. temperature, precipitation, and relative humidity, were aggregated by season along 20-year mean periods: winter (December, January, February), spring (March, April, May), summer (June, July, August), and autumn (September, October, November). Gridded human population data was log10-transformed and divided by polygon area (km2).
Data collection	We retrieved climate, land use, and population data from the Inter- Sectoral Impact Model Intercomparison Project phase 3 (ISIMIP3, https://www.isimip.org/protocol/3/). We requested data on WNV human infections from The European Surveillance System (TESSy) at the European Center for Disease Prevention and Control (ECDC) on https://www.ecdc.europa.eu/en/publications-data/european-surveillance-system-tessy.
Timing and spatial scale	The input data were twofold: 13 years of confirmed WNV human infections across the European continent for the period (2007-2019), as well as climatic, land use, and human population data for a corresponding period (2000-2019).
Data exclusions	There was no data exclusion.
Reproducibility	The study can be reproduced using the R scripts available at https://github.com/sdellicour wnv_enm_europe.<br>In this study, we trained ten replicate models on present-day data retrieved and projected in the past considering either the historical climate or its respective counterfactual. These replicates assure the reproducibility of the findings.
Randomization	There was no group allocation performed in our study.
Blinding	This study does not involve human subjects thus blinding was not necessary. Data on WNV human infections is officially reported to the European Center for Disease Prevention and Control (ECDC).

Did the study involve field work? ☐ Yes ☒ No

Reporting for specific materials, systems and methods

We require information from authors about some types of materials, experimental systems and methods used in many studies. Here, indicate whether each material, system or method listed is relevant to your study. If you are not sure if a list item applies to your research, read the appropriate section before selecting a response.

Materials & experimental systems

n/a	Involved in the study
☒	☐ Antibodies
☒	☐ Eukaryotic cell lines
☒	☐ Palaeontology and archaeology
☒	☐ Animals and other organisms
☒	☐ Clinical data
☒	☐ Dual use research of concern
☒	☐ Plants

Methods

n/a	Involved in the study
☒	☐ ChIP-seq
☒	☐ Flow cytometry
☒	☐ MRI-based neuroimaging
